# Late-Stage Downregulation of miR-138-5p Promotes Colorectal Cancer Progression

**DOI:** 10.3390/ijms27083380

**Published:** 2026-04-09

**Authors:** Hibah Shaath, Radhakrishnan Vishnubalaji, Nehad M. Alajez

**Affiliations:** 1 Translational Oncology Research Center (TORC), Qatar Biomedical Research Institute (QBRI), Hamad Bin Khalifa University (HBKU), Qatar Foundation (QF), Doha P.O. Box 34110, Qatar; hshaath@hbku.edu.qa (H.S.); vbradhakrishnan@hbku.edu.qa (R.V.); 2 College of Health & Life Sciences, Hamad Bin Khalifa University (HBKU), Qatar Foundation (QF), Doha P.O. Box 34110, Qatar

**Keywords:** colorectal cancer, microRNA, miR-138-5p, tumor suppressor, cancer progression, gene regulation, cell growth, migration, organoids, therapeutic targets

## Abstract

Colorectal cancer (CRC) persists as a significant public health burden due to its high morbidity and mortality rates worldwide, yet the molecular events that govern its initiation and progression remain incompletely understood. We recently conducted microRNA (miRNA) profiling and identified multiple dysregulated miRNAs in CRC compared to adjacent normal tissue. Among those, miR-138-5p emerged as a potential tumor suppressor due to its marked downregulation in CRC tissue; however, the stage-specific expression of this miRNA during CRC progression and underlying molecular mechanisms remains to be unraveled. In this study, we performed differential expression profiling of healthy colon, adenomatous polyp (AP), and CRC tissues based on public datasets, revealing significant downregulation of miR-138-5p in CRC compared to controls, but not during the AP stage, suggesting a role in later stages of malignant progression. Forced expression of miR-138-5p in HCT116 and HT-29 CRC models suppressed clonogenic survival, proliferation, and migration while inducing cell death. Additionally, miR-138-5p significantly inhibited tumor formation under three-dimensional culture settings, reinforcing its tumor-suppressive function in a physiologically relevant context. Transcriptomic profiling of miR-138-5p-overexpressing CRC models revealed widespread changes in the pathways related to zinc ion binding, cilium morphogenesis, smoothened signaling, and nuclear transport. Integrated computational and experimental analyses identified 41 potential gene targets, among which *TCF3, UBE2C, EIF4EBP1, LYPLA1*, and *CD44* were validated as potential miR-138-5p-regulated genes. Collectively, these findings establish miR-138-5p as a stage-specific tumor suppressor in CRC, acting through coordinated regulation of oncogenic networks across multiple pathways. Downregulation of miR-138-5p appears to be a late oncogenic event, conferring proliferative, survival, and invasive advantages to tumor cells. Restoration of miR-138-5p or therapeutic targeting of its downstream effectors may represent promising avenues for CRC therapeutic intervention.

## 1. Introduction

Colorectal cancer (CRC) is one of the most frequently diagnosed cancers worldwide and significantly contributes to cancer-associated morbidity and mortality. According to recent GLOBOCAN estimates, CRC ranks third in incidence globally and second in cancer-related mortality [1], despite advances in screening and treatment. Incidence rates are particularly high in developed regions such as North America, Europe, and Australia, but are rising rapidly in developing countries, largely due to the westernization of lifestyle and dietary habits. Risk increases with age, with most cases diagnosed after 50 years, though incidence in younger populations is steadily increasing [2,3,4]. Recent research has increasingly focused on uncovering the molecular changes that contribute to CRC initiation and progression, with the aim of discovering novel diagnostic, prognostic, and therapeutic targets.

CRC develops through a multistep adenoma–carcinoma sequence, a process characterized by the gradual accumulation of genetic mutations and epigenetic changes that drive the transformation of normal mucosa into adenomatous polyps (APs) and eventually invasive CRC [5]. Mutations in the adenomatous polyposis coli (*APC*) gene represent an early event in colorectal tumorigenesis, driving aberrant activation of the Wingless/Integrated (Wnt)/β-catenin signaling pathway and uncontrolled cellular proliferation. Subsequent mutations in Kirsten rat sarcoma viral oncogene homolog (*KRAS*) and activation of the MAPK pathway further enhance tumor cell growth and survival. At more advanced stages, loss of tumor protein P53 (TP53) function and other tumor suppressors facilitates malignant transformation, while additional alterations such as phosphatidylinositol-4,5-bisphosphate 3-kinase catalytic subunit alpha (*PIK3CA*) mutations and disruption of the transforming growth factor-β (TGF-β) signaling pathway, including Mothers against decapentaplegic homolog 4 (SMAD4) inactivation, further contribute to disease progression [6,7].

Epigenetic changes, such as abnormal DNA methylation, histone modifications, and dysregulation of non-coding RNAs (ncRNAs), play a key role in CRC by silencing tumor suppressor genes and altering cellular programs. MicroRNAs (miRNAs), in particular, regulate gene expression through mechanisms such as messenger RNA (mRNA) degradation and translational repression, thereby influencing key cellular processes including proliferation, differentiation, apoptosis, and migration [8]. MiRNAs can function as tumor suppressors or oncogenic drivers (oncomiRs) depending on their specific targets and the cellular context. In CRC, multiple miRNAs have been implicated in disease development and progression, such as miR-21 [9], miR-34a [10], the miR-17-92 cluster [11], miR-143/145 [12], miR-200 family [13,14,15], miR-31, miR-19a [16], and miR-218 [17]. Dysregulation of miRNA expression can result through epigenetic mechanisms such as promoter methylation, copy number changes, transcriptional control, or altered miRNA processing [18,19].

Our earlier study compared miRNA expressions between CRC tissues and adjacent non-tumor tissues, uncovering dysregulation in miRNAs, mRNAs, and long non-coding RNAs (lncRNAs). Notably, our previous analysis revealed persistent downregulation of miR-138-5p in CRC [20]. However, stage-specific expression of miR-138-5p during CRC progression and underlying molecular mechanisms remains to be identified.

Herein, we compared the expression of miR-138-5p in healthy colon, AP and CRC tissues using publicly available datasets and revealed stage-specific miR-138-5p expression. Interestingly, downregulation of miR-138-5p was observed in late CRC, rather than in the AP initiation stages. Reconstitution of miR-138-5p expression led to suppression of key oncogenic phenotypes, including proliferation, migration, and growth under three-dimensional (3D) settings. Transcriptomic profiling and bioinformatics identified several tumor-promoting downstream gene targets for miR-138-5p in CRC. These findings provide new insights into the role of miR-138-5p in CRC progression and potential utilization as therapeutic targets for CRC.

## 2. Results

### 2.1. miR-138-5p Expression During CRC Progression

**Figure 1:**
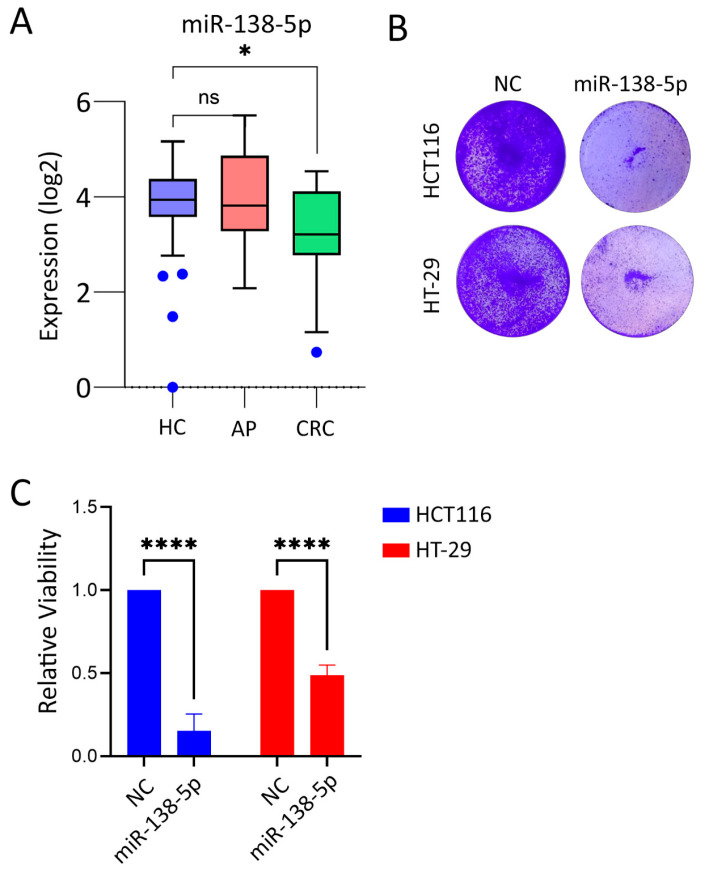
Expression and tumor suppressive function of miR-138-5p in CRC: (**A**) Relative expression of miR-138-5p in healthy controls (HCs, *n* = 32), adenomatous polyps (APs, *n* = 20), and colorectal cancer (CRC, *n* = 20). Center lines represent the median, box limits represent the 25th and 75th percentiles, and whiskers extend to 1.5 times the interquartile range. Individual blue dots indicate outliers as defined by the Tukey method. * *p* < 0.05, n.s. not significant. (**B**,**C**) Effects of miR-138-5p overexpression on CRC cell proliferation. HCT116 and HT-29 cells were transfected with miR-138-5p mimic or negative control mimic. Representative proliferation assay images (**B**) and quantitative analysis (**C**) show a marked reduction in proliferative capacity. Data are presented as mean ± S.D., *n* = 9, **** *p* < 0.0001 from *n* = 3 independent biological replicates. Statistical significance was determined using two-way ANOVA, with *p* < 0.05 considered significant.

Our previous investigation identified miR-138-5p as a downregulated miRNA in CRC compared to normal tissue [17,21]. Comparing its expression in different disease stages, miR-138-5p expression was downregulated in CRC compared to adjacent normal colon tissue; however, no significant differential expression was observed during the AP stage, indicating that miR-138-5p may exert its role at later stages during CRC progression (Figure 1A).

### 2.2. Suppression of CRC Tumorigenicity in Response to miR-138-5p Overexpression Under Two-Dimensional Culture Settings

**Figure 2:**
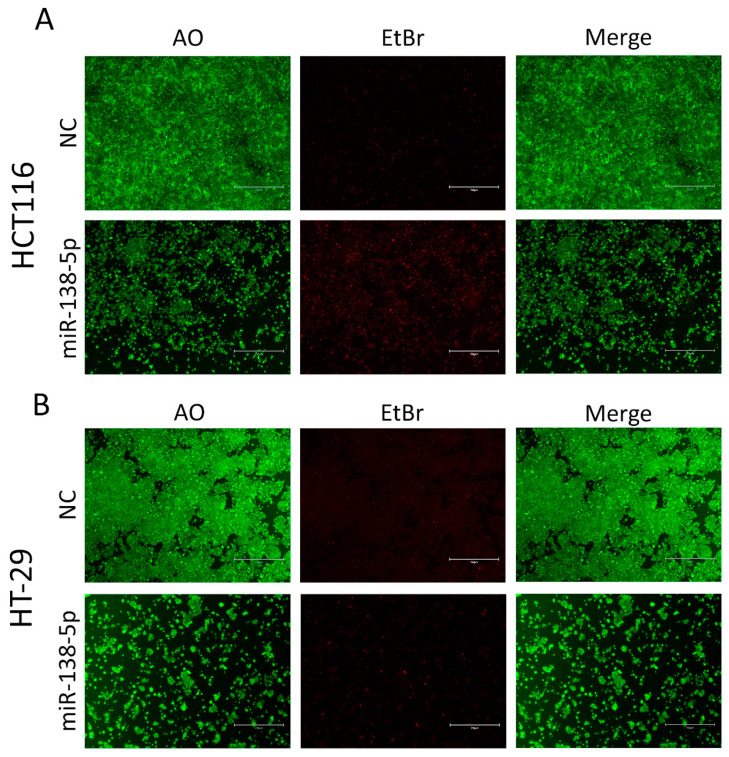
miR-138-5p overexpression reduces cell viability and induces cell death in CRC cells. (**A**,**B**) Representative live–dead acridine orange/ethidium bromide (AO/EtBr) staining in HCT116 and HT-29 cells on day 4 following transfection with miR-138-5p mimic or control mimic, respectively. Increased EtBr-positive staining and condensed morphology, particularly in HCT116, indicate cell death (Scale bars = 750 μm). The experiments presented in this figure were conducted as two independent biological experiments, each performed in technical triplicate, ensuring consistency and reproducibility at the technical level.

To investigate the biological function of miR-138-5p in CRC, HCT116 and HT-29 cells were transfected with a miR-138-5p mimic or a negative control mimic (Figure S1) and subsequently evaluated for cell survival and proliferative capacity. Exogenous expressions of miR-138-5p significantly inhibited proliferation in both the HCT116 and HT-29 cells compared to the control-transfected cells (Figure 1B). Quantitative analysis showed a marked reduction in proliferative potential, ranging from 45% to 80% in the HCT116 and HT-29 cells, respectively (Figure 1C). To investigate the mode of cell death and associated morphological changes induced by miR-138-5p overexpression, we performed a live–dead AO/EtBr (acridine orange/ethidium bromide) staining assay for each condition (Figure 2A,B). The results were consistent with our proliferation assay findings. In both CRC cell models, the cells transfected with the non-targeting control exhibited normal morphology and minimal cell death. In contrast, miR-138-5p overexpression markedly reduced cell viability, evident as decreased cell number after 4 days due to cell death and inhibited proliferation. Additionally, we observed more morphologically condensed cells, with a substantially higher proportion staining positive with EtBr, particularly in HCT116, indicative of cell death, as shown in the second panel and merged images (Figure 2B).

### 2.3. miR-138-5p Suppresses Organoid Formation Under Three-Dimensional (3D) Culture Conditions

**Figure 3:**
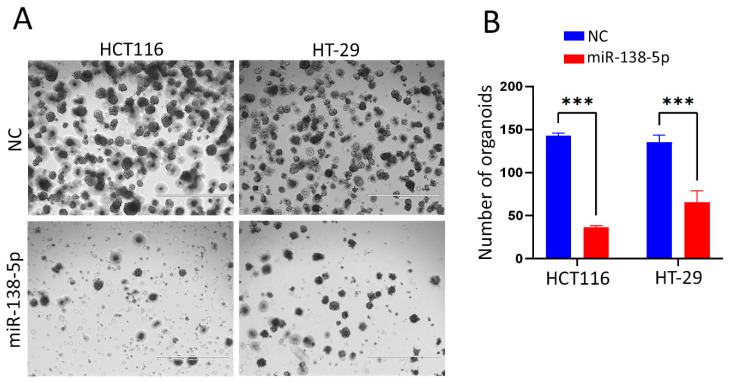
miR-138-5p inhibits three-dimensional (3D) organoid formation in colorectal cancer models. HCT116 and HT-29 cells transfected with miR-138-5p mimic or negative control were embedded in Matrigel and cultured under 3D conditions. (**A**) Representative images illustrating the suppression of HCT116 (left panel) and HT-29 (right panel) growth under 3D conditions in response to exogenous expression of miR-138-5p. (Scale bar = 1000 μM). (**B**) Quantification data for organoid numbers are presented as mean ± S.D., *n* = 2. *** *p* < 0.0005 from *n* = 2 independent biological replicates. Statistical significance was determined using two-way ANOVA, with *p* < 0.05 considered significant. *** *p* < 0.0005.

miR-138-5p overexpression was evaluated for its impact on the 3D growth capacity of CRC cells. The HCT116 and HT-29 cells transfected with miR-138-5p mimic or negative control were embedded in Matrigel and cultured under 3D conditions. As shown in Figure 3A, the miR-138-5p-transfected cells generated markedly fewer organoids compared with controls in both cell lines. Quantification confirmed a significant reduction in organoid number (Figure 3B), consistent with an inhibitory effect of miR-138-5p on CRC cell growth in both two- and three-dimensional models.

### 2.4. Suppression of Cell Migration in Response to miR-138-5p Overexpression

**Figure 4:**
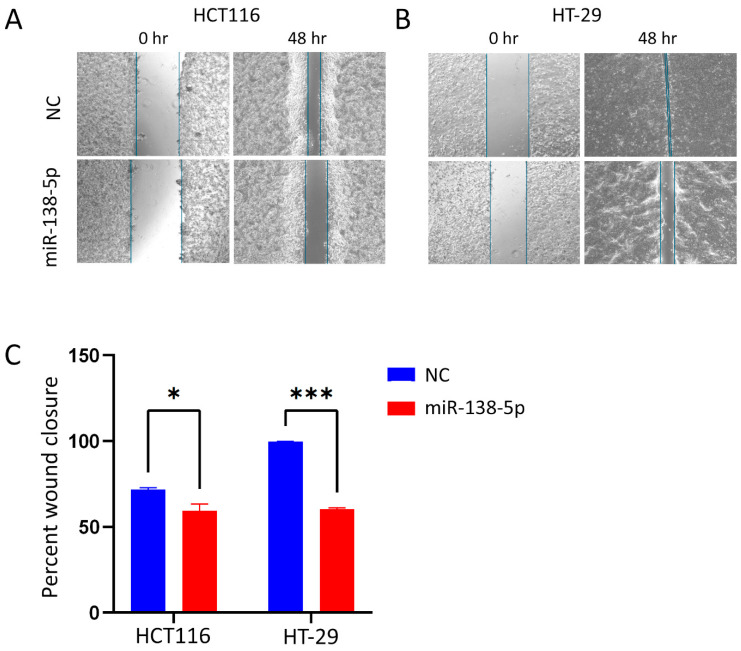
miR-138-5p suppresses cell migration of CRC cells. (**A**,**B**) Representative wound healing assay images in HCT116 and HT-29 cells transfected with miR-138-5p mimic or control mimic, respectively. (**C**) Quantitative analysis of wound closure in miR-138-5p-overexpressing compared to cells transfected with control miRNA mimic. Blue lines indicate the wound margins and are included to clearly delineate the wound area at 0 h and 48 h (Scale bars = 750 μm). Data are presented as mean ± S.D., from *n* = 2 independent biological replicates. Statistical significance was determined using two-way ANOVA, with *p* < 0.05 considered significant. * *p* < 0.05; *** *p* < 0.0005.

We next evaluated the effect of miR-138-5p overexpression on cell migratory potential. Wound healing assays revealed a decrease in migration in both the HCT116 and HT-29 cells following miR-138-5p re-expression (Figure 4A,B). Quantitative analysis revealed that both cell lines exhibited a significant reduction in their ability to close the scratch wound in monolayer cultures (Figure 4C).

### 2.5. RNA-Seq-Based Transcriptomic Characterization and Pathway Enrichment of miR-138-5p-Transfected CRC Cells

**Figure 5:**
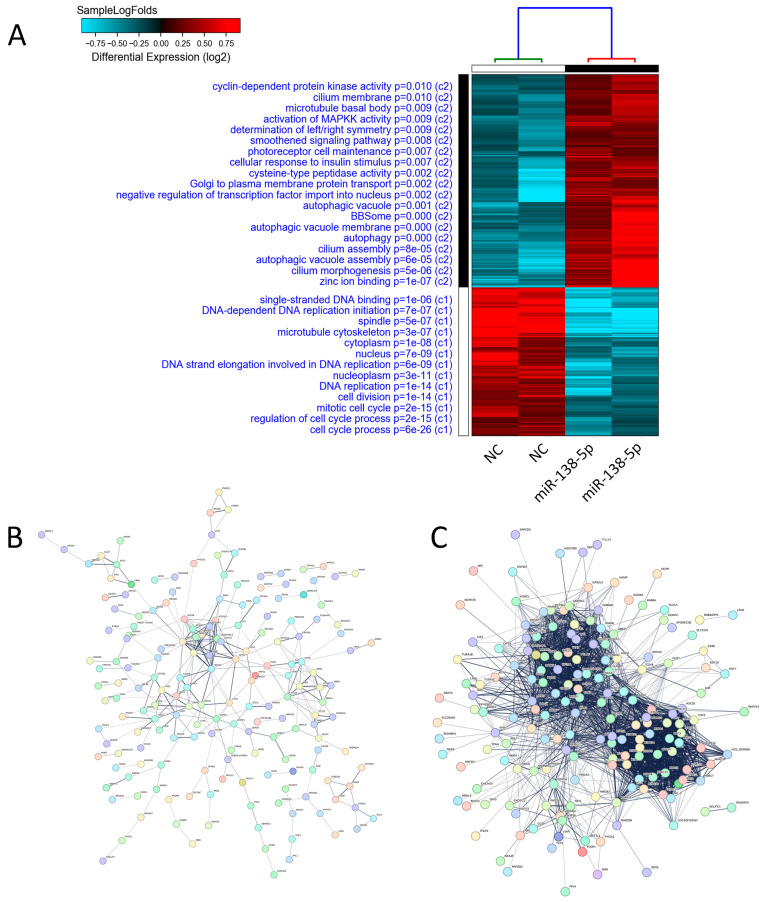
Transcriptomic analysis and pathway enrichment in miR-138-5p-overexpressing CRC cells: (**A**) RNA-Seq analysis performed 72 h post-transfection showing clear clustering of miR-138-5p-overexpressing cells distinct from control mimic-transfected cells based on mRNA expression. Enriched gene ontology (GO) pathways are shown on the left side. (**B**) STRING PPI network of upregulated genes in miR-138-5p-overexpressing CRC cells. (**C**) STRING PPI network of downregulated genes in miR-138-5p-overexpressing CRC cells.

Global transcriptomic changes associated with miR-138-5p overexpression were examined by RNA sequencing. The HCT116 and HT-29 cells were collected three days after transfection, and total RNA was isolated for library preparation and sequencing. Differential expression analysis demonstrated distinct transcriptional profiles separating the miR-138-5p-transfected cells from the negative control cells (Figure 5A and Table S1). Functional enrichment analysis identified significant overrepresentation of the pathways related to zinc ion binding, cilium assembly, autophagy, smoothened signaling pathways, and many others, while the processes related to cell cycle were suppressed (Figure 5A). STRING PPI network analysis of upregulated genes revealed significant enrichment in biological processes related to autophagy, organelle assembly, cellular catabolic processes, and cilium organization (Figure 5B and Table S2). Conversely, PPI network analysis of genes downregulated upon miR-138-5p overexpression demonstrated enrichment in nucleosome assembly, chromatin assembly, nucleosome organization, protein–DNA complex organization, chromatin remodeling, chromosome organization, and cell-cycle-related processes, including the mitotic cell cycle (Figure 5C and Table S3).

### 2.6. Identification of Potential Gene Targets for miR-138-5p in CRC

**Figure 6:**
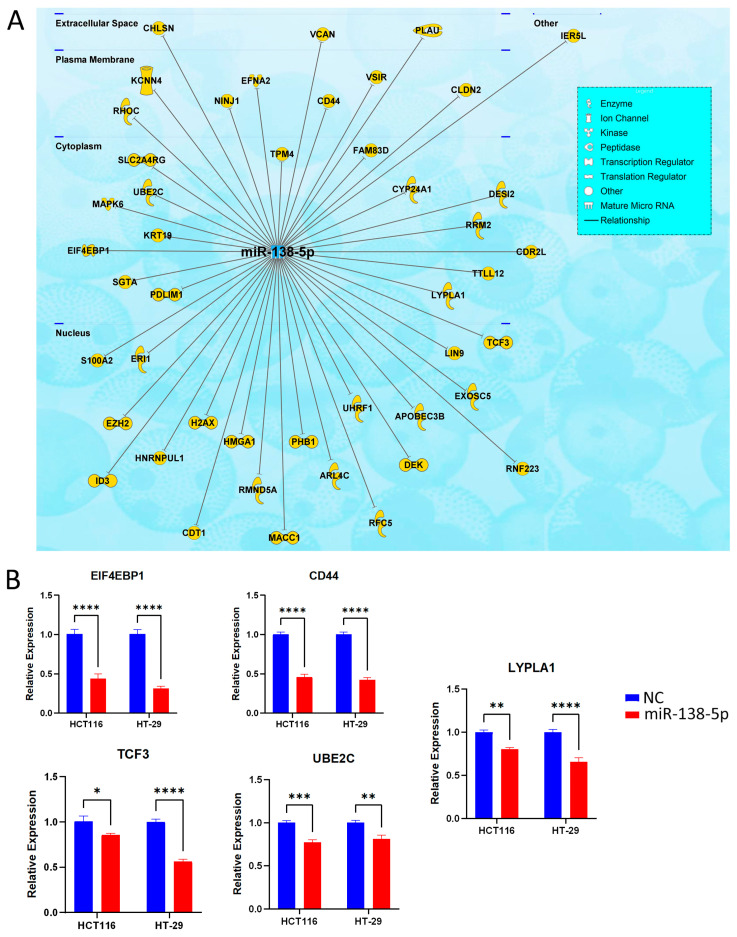
Identification and validation of potential miR-138-5p targets in CRC: (**A**) Integrated IPA microRNA target filter analysis and experimental screening identifying 41 putative miR-138-5p targets and their cellular localization (nuclear, cytoplasmic, plasma membrane, and extracellular compartments). (**B**) Validation of five key gene targets (TCF3, UBE2C, EIF4EBP1, LYPLA1, and CD44) using RT-qPCR in HCT116 and HT-29 cells following miR-138-5p overexpression. Data are presented as mean ± S.D., *n* = 6, from *n* = 3 independent biological replicates. Statistical significance was determined using two-way ANOVA, with *p* < 0.05 considered significant. * *p* < 0.05; ** *p* < 0.005; *** *p* < 0.0005; **** *p* < 0.00005.

To further explore potential mRNA targets of miR-138-5p in CRC cells, we combined experimental validation with computational predictions, identifying 41 potential gene targets using the IPA microRNA target filter analysis (Figure 6A and Table S4). Our analysis identified various gene targets for miR-138-5p, including enzymes involved in transcription and translation regulation, and transporters in the nucleus, cytoplasm, plasma membrane and extracellular space, as shown in Figure 6A. Selected targets including transcription factor 3 (TCF3), ubiquitin-conjugating enzyme E2 C (UBE2C), Eukaryotic Translation Initiation Factor 4E Binding Protein 1 (EIF4EBP1), lysophospholipase 1 (LYPLA1), and cell surface glycoprotein CD44, were validated employing RT-qPCR, demonstrating significant downregulation in both the HCT116 and HT-29 CRC cell lines upon forced miR-138-5p expression (Figure 6B).

## 3. Discussion

Our study adds to a growing body of evidence that miR-138-5p is a key tumor suppressor in CRC, lost during malignant progression and exerting multifaceted inhibitory effects on CRC cell proliferation, migration, and survival. We confirmed that miR-138-5p is downregulated in CRC compared to healthy controls, with little to no change in APs, suggesting that its dysregulation is associated with late stages of tumorigenesis and malignant transition, rather than early adenoma formation. Consistent with prior studies, lower expression of miR-138-5p correlated with advanced tumor stage, lymph node metastasis, elevated Programmed death-ligand 1 (PD-L1) levels, and reduced overall survival, supporting its role as a tumor-suppressive miRNA [22,23]. A limitation of this study is that the stage-specific expression pattern was derived from a single public dataset, and the lack of independent datasets encompassing normal mucosa, adenomatous polyps, and colorectal cancer, as well as the absence of survival analyses in stage-matched cohorts, limited the ability to fully establish the clinical significance of miR-138-5p across disease progression.

Functionally, restoration of miR-138-5p expression reduced clonogenic survival, suppressed proliferation, and impaired migration of CRC cell lines. These findings are concordant with earlier studies reporting that miR-138-5p negatively regulates invasion and epithelial–mesenchymal transition (EMT) in multiple tumor contexts [24]. Live–dead staining confirmed that miR-138-5p overexpression impaired cell viability, consistent with the growth suppression observed in colony formation assays. Notably, evidence of cell death in the CRC cells suggests that miR-138-5p can influence both survival and death pathways. Recent reports highlighted that downregulation of miR-138-5p in CRC leads to mitochondrial calcium uniporter (MCU) upregulation and increased reactive oxygen species (ROS) production associated with CRC progression [25,26]. Furthermore, miR-138-5p has been shown to target Human Telomerase Reverse Transcriptase (hTERT), an important oncogene in multiple cancers, including in CRC, delaying proliferation and increasing apoptosis when overexpressed in cell lines [27].

Our organoid assays further strengthen these observations, as forced expression of miR-138-5p profoundly inhibited 3D organoid formation. Organoids more closely recapitulate tumor architecture and microenvironmental interactions than monolayer cultures, making these results particularly relevant for translational applications [28]. Together, these functional data support the hypothesis that loss of miR-138-5p expression contributes to tumor initiation and progression by enabling sustained proliferation, survival, and invasive behavior.

Transcriptomic profiling of miR-138-5p highlighted enrichment of processes including zinc ion binding, cilium assembly, smoothened signaling, and regulation of nuclear transport, underscoring the pleiotropic effects of miR-138-5p on cellular function. Importantly, several validated mRNA targets were identified and confirmed to be repressed following miR-138-5p overexpression. These targets included TCF3, a transcription factor involved in EMT and stemness [29], which was found to associate with miR-138-5p in the nucleus (Figure 6A). Additional cytoplasmic targets were UBE2C, a ubiquitin-conjugating enzyme required for cell-cycle progression [30]; EIF4EBP1, a regulator of cap-dependent translation that binds directly to eIF4E, the rate-limiting component of the complex that recruits the 40S ribosomal subunit to the 5′ end of mRNAs [31]; and LYPLA1; an α/β-hydrolase family member with both depalmitoylating and lysophospholipase activity [32,33], which contributes to the regulation of protein localization [34]. While these three proteins interact with miR-138-5p in the cytoplasm, CD44, a cell surface marker linked to CRC stemness and invasion, was also found to be regulated by miR-138-5p in this study. Notably, CD44 overexpression increased spheroid-formation efficiency and cell proliferation while reversing the EMT phenotype [35]. The diversity of these targets illustrates how miR-138-5p loss may disrupt multiple signaling axes simultaneously, driving tumor aggressiveness.

A well-characterized regulatory interaction has been reported, wherein miR-138-5p directly targets PD-L1 in CRC [22], thereby not only affecting tumor cell biology and progression, but also potentially modulating immune evasion via association with tumor-infiltrating lymphocytes [36]. Previous studies show that low miR-138-5p/high PD-L1 expression correlates with poorer outcome in CRC patients [22], non-small-cell lung cancer (NSCLC) [37,38], and breast cancer [39], where overexpression of miR-138-5p led to reduced PD-L1 expression and tumor growth. Our validated set of targets fits well with known oncogenic pathways and expands the repertoire of miR-138-5p’s suppressive functions.

Another emerging area is epigenetic regulation of miR-138-5p. Recent studies found that promoter methylation of the miR-138-5p gene is elevated in CRC patients, both in tissues and in plasma, and this methylation correlates with reduced miR-138-5p expression and increased PD-L1 expression [40]. Our recent study on another miRNA corroborated this finding, where miR-218-5p, embedded within the Slit Guidance Ligand 2 (SLIT2) and Slit Guidance Ligand 3 (SLIT3) introns on chromosome 4 and chromosome 5, respectively, revealed epigenetic silencing through promoter hypermethylation in CRC cell models [17].

While our integrative transcriptomic and functional analyses identified multiple candidate targets of miR-138-5p, we acknowledge that protein-level validation was not performed in the current study. Given that miRNAs primarily exert their regulatory effects through translational repression and/or mRNA degradation, future studies incorporating protein-based assays (e.g., Western blotting or proteomic profiling) will be essential to confirm the direct impact of miR-138-5p on these targets. In addition, luciferase reporter assays will be necessary to validate direct miRNA–mRNA interactions by confirming binding to the 3′ untranslated regions (3′UTRs) of candidate target genes. Together, these approaches will provide a more comprehensive understanding of the underlying molecular mechanisms mediated by miR-138-5p.

## 4. Materials and Methods

### 4.1. Sample Preparation, Sequencing, and Bioinformatics Analysis

Publicly available small RNA sequencing data comprising healthy control, AP, and CRC tissues were obtained from the PRJNA673192 dataset. Sequence analysis was conducted using CLC Genomics Workbench with the latest miRBase annotations [17]. Raw mature miRNA read counts were normalized using the trimmed mean of M values (TMM) method. Normalized expression values for miR-138-5p were extracted and compared across the HC, AP, and CRC groups.

### 4.2. Cell Culture

The HCT116 and HT-29 human CRC cell lines were maintained in Dulbecco’s Modified Eagle Medium (DMEM; Gibco, Thermo Fisher Scientific, Waltham, MA, USA; Cat. No. 11965-092) supplemented with 10% fetal bovine serum (FBS; Gibco, Thermo Fisher Scientific, Waltham, MA, USA; Cat. No. 16000-044) and 1% penicillin–streptomycin solution (Gibco, Thermo Fisher Scientific, Waltham, MA, USA; Cat. No. 15140-122). The cells were cultured as adherent monolayers at 37 °C in a humidified incubator containing 5% CO_2_.

### 4.3. miR-138-5p Mimic Transfection and Quantification

The cells were transfected with hsa-miR-138-5p mirVana™ mimic (Ambion, Thermo Fisher Scientific, Waltham, MA, USA; Cat. No. 4464066) or mirVana™ negative control mimic #1 (Ambion, Thermo Fisher Scientific, Waltham, MA, USA; Cat. No. 4464058). Mimics (final concentration 30 nM) and Lipofectamine™ 2000 (1.5 μL; Invitrogen, Thermo Fisher Scientific, Waltham, MA, USA; Cat. No. 52758) were diluted separately in Opti-MEM and incubated for 20 min at room temperature to allow complex formation. The transfection mixture (200 µL) was added to 800 µL of medium containing 0.168 × 10^6^ cells/mL. After 24 h, fresh medium was added. For miRNA reverse transcription, 10 ng of total RNA was used for first-strand cDNA synthesis using the miRCURY LNA RT Kit (QIAGEN, Hilden, Germany; Cat. No. 339340). The relative miRNA expression levels were quantified using hsa-miR-138-5p miRCURY LNA miRNA PCR Assay (QIAGEN, Hilden, Germany; ID YP00206078) in combination with the miRCURY LNA SYBR^®^ Green PCR Kit (QIAGEN, Hilden, Germany; Cat. No. 339345). The relative expression levels were calculated using the 2^−ΔΔCt^ method, with SNORD48 used as the endogenous control for normalization.

### 4.4. Clonogenic Proliferation Assay

Cell proliferation was evaluated by a colony formation assay. The transfected cells were seeded into 12-well plates and cultured for four days. The colonies were fixed and stained with Crystal Violet (Sigma-Aldrich, St. Louis, MO, USA), air-dried, and imaged. For quantification, dye was solubilized in 10% sodium dodecyl sulfate (SDS) (Sigma-Aldrich, St. Louis, MO, USA), and absorbance was measured in duplicate using a NanoQuant Plate with an Infinite M200 Pro reader (Tecan; Männedorf, Switzerland).

### 4.5. Assessment of Cell Death by AO/EtBr Staining

On day 4 post-transfection, the cells were washed with PBS and incubated with AO/EtBr solution (100 mg/mL each) (AO/EtBr, Sigma Aldrich, St. Louis, MO, USA) for 2 min. Fluorescent images were captured using an Olympus IX73 microscope (Olympus Corporation, Tokyo, Japan). AO staining was used to assess nuclear morphology, while EtBr-positive cells indicated cell death.

### 4.6. Three-Dimensional Organoid Culture

The transfected cells were collected on day 4 and suspended in Matrigel^®^ Matrix (Corning Inc., Corning, NY, USA; Cat. No. 354234). Cell–Matrigel mixtures were plated as three domes in 6 cm dishes and allowed to polymerize for 20 min at 37 °C. The domes were overlaid with DMEM and cultured. Organoid formation was monitored, and images were acquired on day 7 using the EVOS™ Cell Imaging System (Thermo Fisher Scientific, Waltham, MA, USA). Organoids were quantified using OpenCFU software (v3.9.0) [41].

### 4.7. Scratch Wound Healing Assay

For migration analysis, the transfected cells were reseeded into 12-well plates at confluence. A linear scratch was introduced using a 200 µL pipette tip. After washing with PBS, fresh medium was added. Images were captured at 0 and 48 h using phase-contrast microscopy. Wound closure was quantified by measuring the wound area with ImageJ software (ImageJ version 1.54q (Java 1.8.0_431, 64-bit)) [42].

### 4.8. RNA Isolation and Next-Generation Sequencing

Total RNA was isolated from the cells 72 h after transfection. Libraries were prepared using the TruSeq Stranded Total RNA Library kit (Illumina, San Diego, CA, USA) and sequenced on the Illumina NextSeq 2000 platform, generating approximately 50 million paired-end reads (2 × 75 bp) per sample [43]. Two independent biological replicates were included for each condition. FASTQ files were aligned to the hg38 reference genome using CLC Genomics Workbench (v21.0.5). Gene expression levels were calculated as transcripts per million (TPM). Differential expression analysis was performed using AltAnalyze (v2.1.3) with a ≥2-fold change threshold and FDR-adjusted *p*-value cutoff. KEGG pathway enrichment was conducted using STRING (v12.0) [44].

### 4.9. miRNA Target Identification and RT-qPCR Validation

Differentially expressed genes were analyzed using the Ingenuity Pathway Analysis miRNA Target Filter to predict miR-138-5p regulatory networks. The selected targets were validated by RT-qPCR using gene-specific primers (Table 1) and PowerUp™ SYBR Green Master Mix on a QuantStudio™ 6 Flex system. Expression levels were normalized to β-actin (ACTB) and calculated using the 2^−ΔΔCt^ method relative to control cells [45].

## 5. Conclusions

Our findings reinforce that miR-138-5p downregulation is a hallmark of CRC progression, and that restoration of this miRNA impairs multiple malignant phenotypes. Our study provides mechanistic and functional evidence that miR-138-5p suppresses CRC progression through coordinated regulation of oncogenic drivers at different cellular compartments. Downregulation of miR-138-5p appears to be a late event in colorectal tumorigenesis, conferring selective growth and survival advantages to malignant cells. Given its broad impact, therapeutic strategies aimed at restoring miR-138-5p activity or pharmacologically targeting its downstream effectors hold potential as future CRC interventions. Further in vivo validation and patient-derived studies will be essential to explore these therapeutic opportunities.

## Figures and Tables

**Table 1:** SYBR green and methylation primer sequences used in current study.

Names	Primer Sequences (5′-3′)
ACTB_F	GGCACCCAGCACAATGAAG
ACTB_R	CCGATCCACACGGAGTACTTG
TCF3_F	AGAAGCCCCAGACCAAACTG
TCF3_R	GGATTCAGGTTCCGCTCTCG
LYPLA1_F	TGTGAGCTGAGGCGGTGTA
LYPLA1_R	ATGCAGGAAAATCACCGCAG
UBE2C_F	GTTCCTGTCTCTCTGCCAACG
UBE2C_R	GTCTGATTCAGGGAAGGCAGAA
EIF4EBP1_F	CTATGACCGGAAATTCCTGATGG
EIF4EBP1_R	CCCGCTTATCTTCTGGGCTA
CD44_F	GCAGTCAACAGTCGAAGAAGG
CD44_R	TGTCCTCCACAGCTCCATT

## Data Availability

Data are provided as supplementary data. Additional data are available upon request from the corresponding authors.

## Supplementary Materials

The following supporting information can be downloaded at https://www.mdpi.com/article/10.3390/ijms27083380/s1.

## Abbreviations

The following abbreviations are used in this manuscript:

**Abbreviation**	**Full Term**
AO/EtBr	Acridine Orange/Ethidium Bromide
AP	Adenomatous Polyps
CD44	Cluster of Differentiation 44
CFU	Colony Formation Unit (assay)
CLC	CLC Genomics Workbench
CRC	Colorectal Cancer
EIF4EBP1	Eukaryotic Translation Initiation Factor 4E Binding Protein 1
eIF4E	Eukaryotic Translation Initiation Factor 4E
HC	Healthy Controls
HT-29	Human Colorectal Adenocarcinoma Cell Line HT-29
HCT116	Human Colorectal Carcinoma Cell Line HCT116
LYPLA1	Lysophospholipase 1
miRNA	MicroRNA
NC	Negative Control
RNA-seq	RNA Sequencing
TCF3	Transcription Factor 3 (E2A, immunoglobulin enhancer-binding factor E12/E47)
UBE2C	Ubiquitin-Conjugating Enzyme E2 C
