# Supplementary material for: Late-Stage Downregulation of miR-138-5p Promotes Colorectal Cancer Progression
Source: Int J Mol Sci. 2026 Apr 9;27(8):3380. doi: 10.3390/ijms27083380 (PMC13116096; doi:10.3390/ijms27083380)
Supplement: Supplementary file 1 [file ijms-27-03380-s001.zip › ijms-4172683-supplementary revised/Figure S1.pdf]

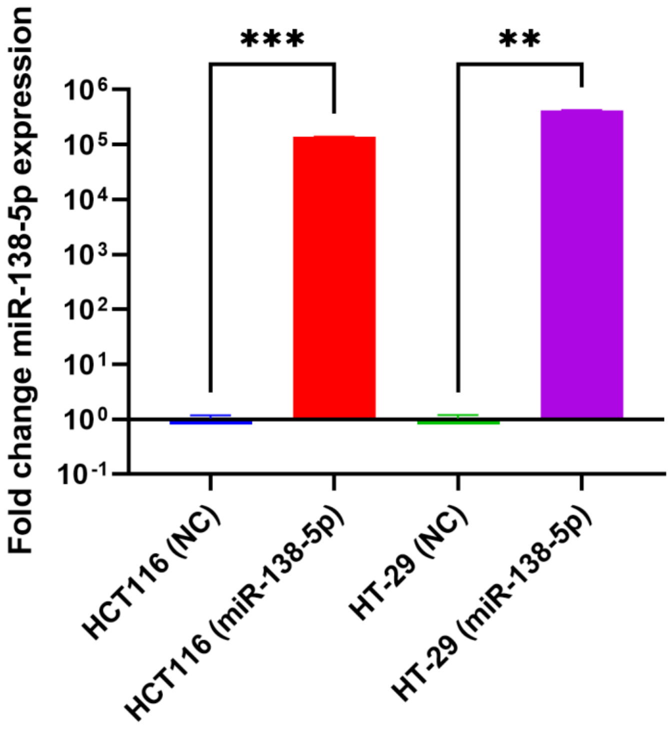

Figure S1. Expression of miR-138-5p in transfected HCT116 and HT-29 cells. Mature miR-138-5p was detected using miRCURY LNA miRNA PCR Assays at 48 hours post-transfection with negative control (NC) or miR-138-5p mimics. Data are presented as mean ± SEM (n = 3).
